# Capsular Management in Hip Arthroscopy: An Anatomic, Biomechanical, and Technical Review

**DOI:** 10.3389/fsurg.2016.00013

**Published:** 2016-03-04

**Authors:** Benjamin D. Kuhns, Alexander E. Weber, David M. Levy, Asheesh Bedi, Richard C. Mather, Michael J. Salata, Shane J. Nho

**Affiliations:** ^1^Department of Orthopedic Surgery, Division of Sports Medicine, Hip Preservation Center, Rush University Medical Center, Rush Medical College of Rush University, Chicago, IL, USA; ^2^Department of Orthopedic Surgery, Division of Sports Medicine, University of Michigan Health System, Ann Arbor, MI, USA; ^3^Department of Orthopedic Surgery, Duke University Hospital, Durham, NC, USA; ^4^Department of Orthopedic Surgery, University Hospitals, Cleveland, OH, USA

**Keywords:** hip arthroscopy, hip capsule, hip instability, hip joint, capsulotomy technique, capsular repair

## Abstract

Hip arthroscopy has become an increasingly utilized surgical technique for the treatment of the young, active patients with hip pain. The clinical outcomes of hip arthroscopy in this patient population have been largely successful; however, there is increasing interest in the contribution of hip capsule in postoperative clinical and functional outcomes. The structure and function of the normal hip capsule will be reviewed. Capsular contributions to hip stability will be discussed in the setting of hip arthroscopy with an emphasis on diagnosis-based considerations. Lastly, clinical outcomes following hip arthroscopy will be discussed as they relate to capsular management.

## Introduction

In recent years, hip arthroscopy has become the surgical technique of choice for the treatment of a variety of symptomatic disorders of the hip, including femoroacetabular impingement (FAI). This meteoric rise in hip arthroscopy is in large part due to the minimally invasive nature of the surgical approach. When indicated, hip arthroscopic procedures have demonstrated excellent short- and mid-term functional outcomes and high satisfaction and return to activity rates beginning with patients as young as 11 years of age (1). The ability to successfully treat a spectrum of hip disorders is limited by the arthroscopic exposure of the offending pathology whether it is in the central, peripheral, or peritrochanteric compartments. Surgical management of the hip capsule is crucial to provide exposure to the aforementioned regions during arthroscopy, and described techniques include capsulectomy, capsulotomy, and capsulotomy with repair. The selected approach should consider various factors, including patient symptoms, patient baseline general ligament laxity, underlying hip pathology, and surgeon skill level. Failure to consider each of these unique factors for any given surgical case may lead to incomplete treatment of the underlying pathology or postoperative complications related to iatrogenic hip instability. This article will review the anatomy of the hip capsule with an emphasis on structure and function. Diagnosis-based considerations for capsular management will be discussed with an emphasis on surgical techniques and resultant clinical outcomes.

## Hip Joint Anatomy

**Figure 1:**
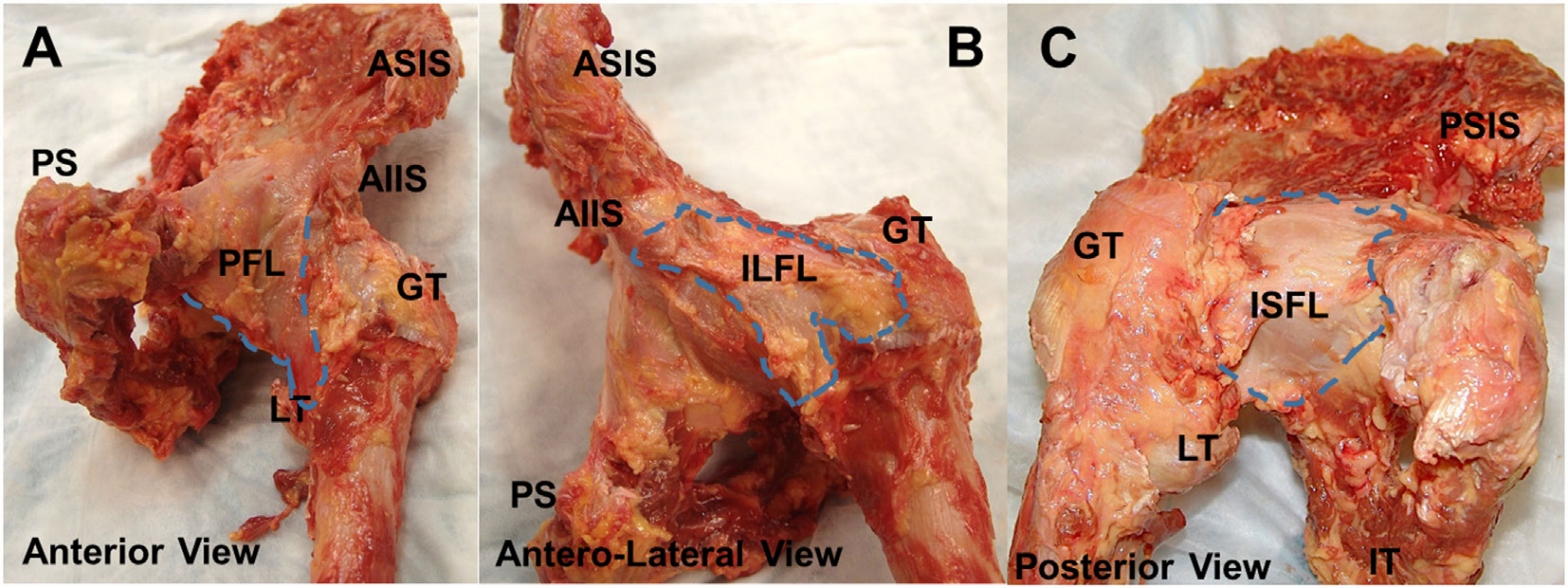
**Anatomy of joint capsule**. Superficial gross anatomy of the hip capsule. The anterior capsule **(A)** is seen with the pubofemoral ligament visualized medially. The ILFL is best appreciated in the anterolateral position **(B)**, and the ischiofemoral ligament can be seen posteriorly **(C)**. AIIS, anteroinferior iliac spine; PFL, pubofemoral ligament; GT, greater trochanter; LT, lesser trochanter; ILFL, iliofemoral ligament; ISFL, ischiofemoral ligament.

The hip capsule is a fibrous structure surrounding the hip joint comprising three external ligaments directed longitudinally as well as internal fibers directed circumferentially. The external ligaments are the iliofemoral ligament (Y ligament of Bigelow; ILFL), ischiofemoral ligament (ISFL), and pubofemoral ligament (PFL). The internal circular fibers of the capsule define the zona orbicularis (ZO) and are lined with synovium encircling the femoral head and neck (2). The native anatomy of these ligaments, including their attachments, thickness, and fiber direction, has been well-documented in numerous reports (Figures 1A–C) (2–6). The hip capsule contains the articulation of the femoral head within the acetabulum, as well as the labrum, transverse acetabular ligament, and ligamentum teres, all of which act to protect and stabilize the joint. Additionally, the capsule is perforated by numerous blood vessels responsible for perfusing the hip joint.

### Ligaments

Knowledge of the anatomy of the hip capsule, as well as its pericapsular musculotendinous attachments, has increased significantly over the past decade. In 2011, Nam et al. illustrated the acetabular origins using precise clock-face positioning, as popularized by Blankenbaker (7, 8). The authors localized the centers of the ILFL, ISFL, and PFL on average to the 1:26, 10:15, and 4:44 positions, respectively. They also found that the origin of the PFL had the smallest insertional footprint running from 4:02 to 5:27, compared to the ILFL, which spanned the 12:35 to 2:18 region and the ISFL between 8:44 and 11:45. This was similar to a study by Telleria et al., who found the PFL, ILFL, and ISFL running from 3:30 to 5:30, 12:45 to 3:00, and 7:45 to 10:30, respectively (9). In a recent cadaveric study, Walters et al. reported the hip capsule to originate 5 mm proximal and medial to the acetabular rim (5). This proximal origin creates a pericapsular recess, which is an important landmark when evaluating capsular laxity on magnetic resonance imaging (MRI) (10).

The ligaments overlap in a way that may be difficult to appreciate distinct capsular contributions arthroscopically. The PFL travels inferoposteriorly under the medial arm of the ILFL and blends with the ISFL near its acetabular insertion (11). The ISFL spirals superolaterally to insert at the base of the greater trochanter anterior to the femoral neck axis (11). Martin et al. described the insertion of the two arms of the ILFL, where the medial arm descends vertically onto the distal intertrochanteric line, and the lateral arm traverses horizontally along the femoral neck to insert onto the anterior greater trochanteric crest (3). The ZO is a distinct structure of the inner capsule comprising circular fibers surrounding the femoral neck. In a study of seven cadavers, Ito et al. found the ZO to increase the stability of the hip in distraction and postulated that it acted as a locking ring around the femoral neck (12).

Capsular thickness is another important feature of the capsular anatomy, especially when choosing where to establish arthroscopic portals. Walters et al. found the capsular origin to be thickest posterosuperiorly (4 mm) and thinnest anteroinferiorly (1.3 mm) (5). Moving distal to its origin, the mid portion of the capsule is thickest superiorly just underneath the attachment of the gluteus minimus (6). This region represents the ILFL and, during arthroscopy, it is the site of interportal capsulotomy between anterolateral and mid-anterior arthroscopic portals. Finally, the capsular insertion is thickest anterosuperiorly and located 26 mm distal to the femoral head–neck junction, creating a large distal intracapsular recess along the femoral neck (5, 8).

Telleria et al. have investigated the arthroscopic applications of our increasingly robust understanding of capsular anatomy. After performing arthroscopy on cadaveric hips, the authors found that an anterolateral (AL) portal generally pierces the ILFL just inside its lateral border, while the mid-anterior portal pierces it medially (9). Thus, the interportal capsulotomy traverses the width of the ILFL and, in this way, may have ramifications on capsular laxity and stability if not properly repaired. The posterolateral portal penetrates the ISFL superolaterally (9). In the peripheral compartment, Telleria et al. found the PFL to be 6 mm lateral to the medial synovial fold (MSF) at the level of the ZO, and the ISFL was 11.7 mm posterior to the lateral synovial fold (LSF). The medial arm of the ILFL was 6 mm lateral to the MSF, and the lateral arm was 3 mm anterior to the LSF (9). It should be noted that the individual ligaments comprising the capsule could not be seen arthroscopically, but rather their discernment required preoperative dissection and border demarcation with 18-gauge needles (9).

### Dynamic Stabilizers

**Figure 2:**
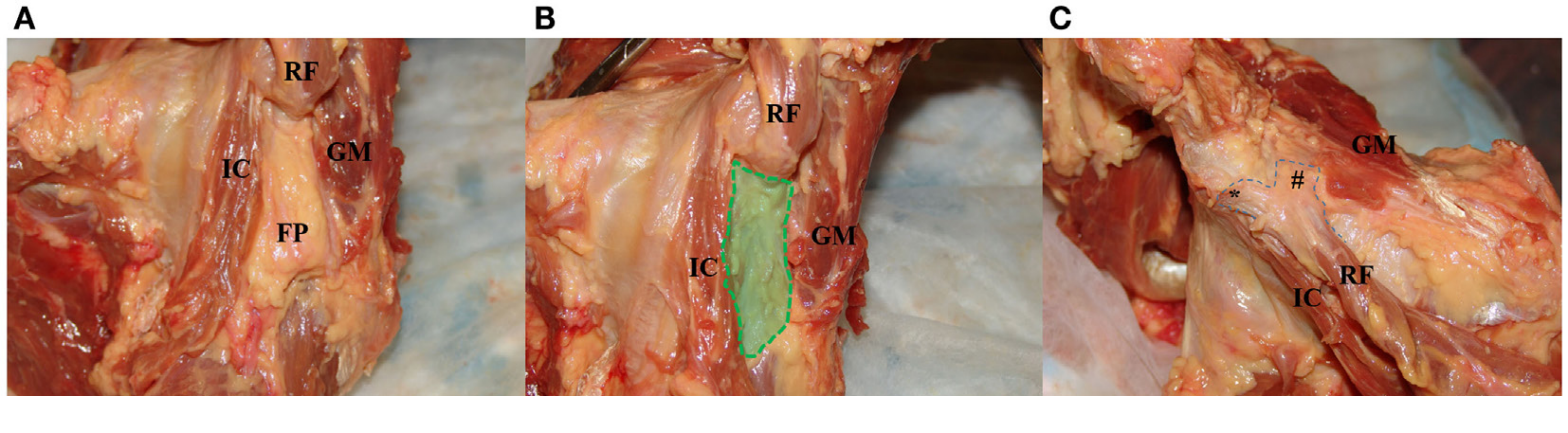
**Images of the dynamic stabilizers of the hip capsule**. **(A)** With the hip capsule positioned laterally, the rectus femoris is resected to reveal a fat pad between the iliocapsularis and gluteus minimus. **(B)** The fat pad is resected to demonstrate the “safe zone” for capsulotomy between the iliocapsularis and gluteus minimus overlying the anterior superior capsule. **(C)** With the hip in the anterior position, the gluteus minimus is partially resected to show the proximal attachment of the rectus femoris to the AIIS (*) and the attachment of its reflected head to the anterior superior capsule (^#^). IC, iliocapsularis; RF, rectus femoris; FP, fat pad deep to the rectus femoris; GM, gluteus minimus.

Muscular contributions to the hip capsule include the iliocapsularis, the indirect head of the rectus femoris, and the gluteus minimus (Figures 2A–C). The iliocapsularis was found to adhere anteromedially (2:30) and had the largest capsular contribution of any musculotendinous structure, originating at the AIIS and inserting on the distal lesser trochanter (4–6). The function of the iliocapsularis is believed to tighten the anterior hip capsule, which can help stabilize the femoral head in dysplastic hips with decreased anterolateral acetabular coverage (5, 13). In an anatomic study comparing dysplastic vs. normal hips, Babst et al. found iliocapsularis hypertrophy in dysplastic hips to support this hypothesis (14). The indirect or reflected head, of the rectus femoris attaches to the capsule near the anterosuperior acetabular rim between 11:30 and 2:00 (4, 5). There is also a fat pad situated between the iliocapsularis attachment and reflected head of the rectus femoris (Figure 2A). The gluteus minimus inserts broadly onto the anterosuperior border of the greater trochanter, and the conjoint tendon and obturator externus run along the posteroinferior capsule (2, 5). While these tendons do not directly insert into the posterior hip capsule, there are adhesions consistently found near the posterior acetabular rim (6). From an arthroscopic standpoint, Walters et al. describe a “stability arc” viewed in the peripheral compartment comprising the superolateral gluteus medius, superomedial reflected head of the rectus femoris, and anteromedial iliocapsularis (Figure 2B) (5). They postulate that the stability arc functions to prevent anterior dislocation and can be used as a guide for a capsulotomy during hip arthroscopy.

### Neurovascular Supply

The capsular blood supply receives contributions from the medial femoral circumflex artery (MFCA), lateral femoral circumflex artery (LFCA), superior gluteal artery (SGA), and inferior gluteal artery (IGA) (15). In a study of 20 cadaveric hips, Kalhor et al. reported that both the MFCA and LFCA give off capsular branches running circumferentially from the distal to proximal capsule, while the IGA and SGA supplied the posterior capsule (15). They also found that many of these branches form a circumferential periacetabular anastomotic ring between distal and proximal vessels. McCormick et al. have shown that the MFCA pierces the periosteum of the posterosuperior femoral neck, medial to the greater trochanter between 10:30 and 12:00 on the neck–shaft axis (16). These authors described the arthroscopic safe zones along the anterior femoral neck for osteochondroplasties and along the middle third of the medial capsule for psoas tenotomies. Kalhor et al. argued that proximal, rather than distal, capsulotomies avoid the femoral head’s vascular supply, as these arteries enter the joint distally (15).

The nociceptive innervation of the capsule was studied histologically by Haversath et al. and found to be evenly distributed throughout the capsule (17). This finding was is in stark contrast with the earlier work of Gerhardt et al. showing an increased concentration of neural fibers in the superolateral capsule (17, 18). However, Haversath et al. had taken samples from diseased arthritic hips during arthroplasty, so their findings of diffuse pain fibers may not be generalizable to patients without arthritis. Overall, precise anatomic knowledge of the hip capsule and surrounding structures can help the arthroscopic surgeon identify intraoperative landmarks and safe zones.

## Capsular Biomechanical Characteristics

Violation of the capsulolabral suction seal is required during arthroscopic hip surgery, and as such has provided the opportunity to clinically study the role of the capsule in overall hip stability. Stability is achieved in part by the ZO spiral configuration acting as a screw home mechanism to stabilize the joint in extension and external rotation (12, 19). This mechanism loosens when the hip is brought into flexion, which may make the joint less stable and prone to injury in this position (3, 19, 20). In a cadaveric study, the anterior capsule was shown to withstand a significant amount of tensile force due in large part to the ILFL acting as the strongest capsular constraint (21). In a range of motion study of 12 cadaveric hips, Martin et al. found that the lateral arm of the ILFL controls external rotation in both flexion and extension, whereas the ISFL constrains internal rotation in these positions (3). They also reported that the ILFL limits internal rotation in extension, which is in contrast to a biomechanical study by Myers et al. that found the ILFL limits external rotation only (3, 22). By applying 5 Nm of external or internal rotation torque in varying degrees of flexion and extension, Myers et al. reported that ILFL resection increases femoral head rotation and anterior translation, while repair of the ILFL reverses these trends. In this same study, Myers et al. reported that a labral repair alone was insufficient to restore the hip to its native range of motion, with complete restoration occurring only after combined labral and capsular repair.

Biomechanical studies have attempted to quantify the degree to which capsulotomies affect femoral head translation, rotation, and axial strain within the acetabulum with and without repair (3, 23). In a cadaveric study of 13 hips after capsulotomy, Bayne et al. reported qualitative increases in anterior femoral head translation in neutral rotation and increased posterior translation with the hip in flexion (23). One biomechanical study investigating the effect of different capsulotomies on hip stability found that the larger the capsulotomy, the greater the degree of hip rotation, and hip capsulectomy and the unrepaired T-type resulted in the greatest degree of rotation. However, complete repair of the capsule decreases hip rotation similar to the unrepaired interportal capsulotomy suggesting that complete repair can improve the rotational profile (24). With these data in mind, it is critical to weigh the benefits of capsulotomy with its risk of iatrogenic instability and to consider repairing the capsule completely. Additional basic science and biomechanical studies are required to further elucidate the role of the capsule in maintaining hip stability in both pre and postoperative FAI populations.

## Hip Instability Subtypes: Traumatic, FAI-Induced, Atraumatic, and Iatrogenic

**Figure 3:**
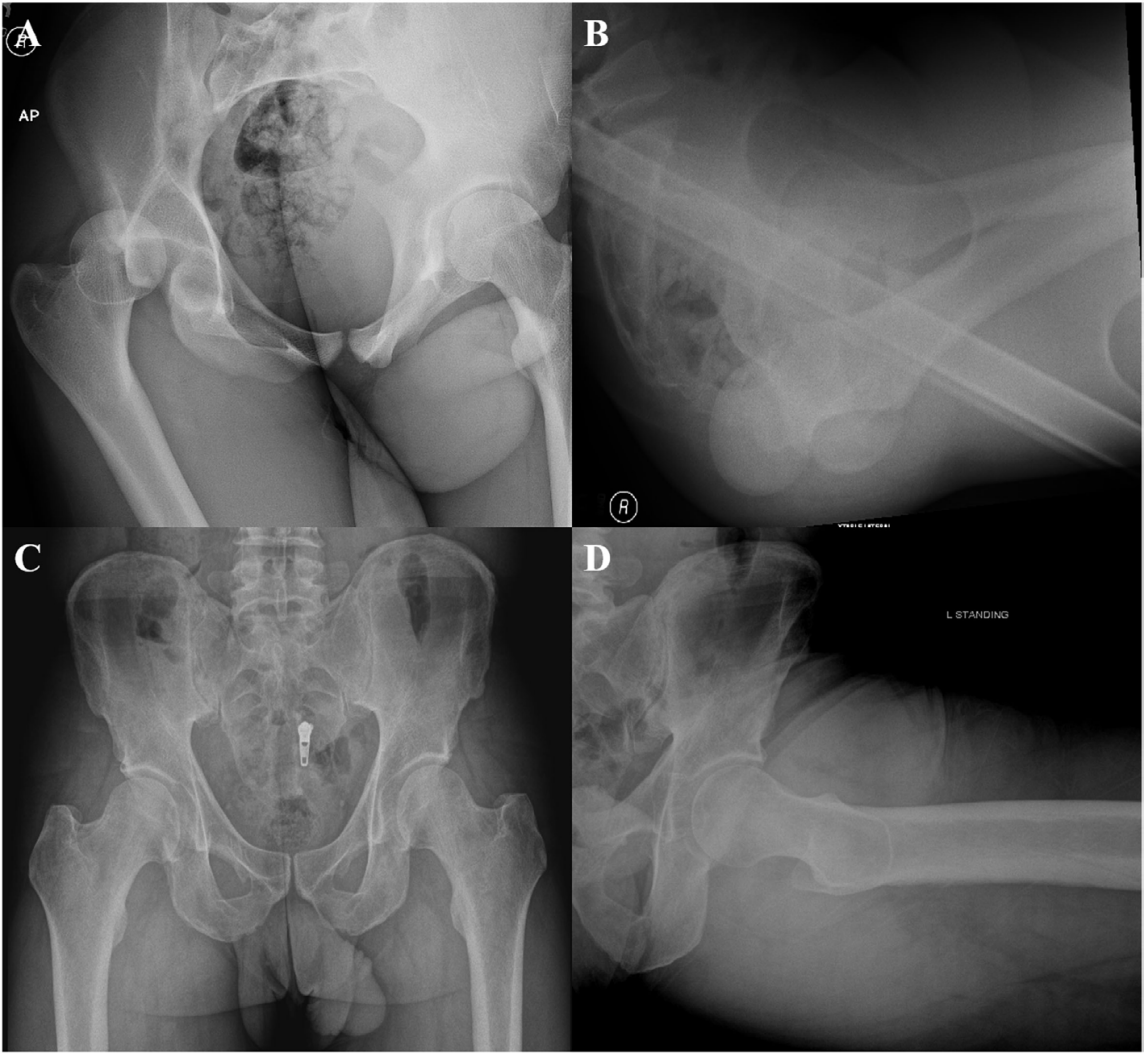
**Anteroposterior (A) and cross table (B) radiographs demonstrating a posterior hip dislocation**. Anteroposterior **(C)** and Dunn **(D)** views demonstrating a borderline dysplastic patient (LCEA 21.6) with a cam deformity (AA 63).

The hip capsule enhances the stability of the hip joint, and capsule-specific pathology has been implicated in hip instability conditions. Hip instability comprises a spectrum of pathological entities ranging from traumatic instability, FAI-induced instability, atraumatic microinstability, and iatrogenic instability (Table 1). Traumatic hip instability itself includes frank dislocations following major trauma, hip subluxation from more minor trauma, and microtrauma following repetitive motion (25). For posterior hip dislocations, the most common injury mechanism is a high energy dashboard injury following a motor vehicle accident in which an axial force is directed against the femoral shaft with the hip in a flexed position (26) (Figures 3A,B). In addition to other injuries outside the hip, this mechanism often produces posterior hip dislocation with a posterior wall fracture, and can include concomitant injury to the labrum, capsule, and chondral surfaces of the femur and acetabulum (27, 28). On the other hand, anterior dislocations occur when a force is directed against an abducted and externally rotated hip with the degree of flexion at the time of injury, determining whether the dislocation is superior or inferior (29). Lower level trauma, such as that seen in athletic competition, can also induce traumatic instability. In a study of 14 traumatic dislocations in professional athletes, Philippon et al. found additional intra-articular pathology on arthroscopy, including labral tears (100%), chondral defects (100%), ligamentum teres tears (78%), and capsular tears (14%) (30). Additionally, in a series of American football players with traumatic posterior subluxation, Moorman et al. report that this cohort presented with the attendant triad of posterior acetabular lip fracture, ILFL disruption, and hemarthrosis (31). Further, sports involving repetitive motion such as golf, hockey, soccer, ballet, and figure skating can induce labral and capsular wear, which promote microinstability resulting in increased femoral head translation within the acetabulum (32).

**Table 1:** **Subtypes of hip instability**.

Types of hip instability	Characteristics
Traumatic	Two types: (1) high impact event with frank joint dislocation; (2) hip subluxation resulting from microtrauma of repetitive supraphysiologic motion
Atraumatic	Associated with the borderline dysplasia and ligamentous laxity
FAI related	Posterior subluxation in the setting of FAI
Iatrogenic	Presents as gross dislocation (rare) and could be a mechanism for postoperative pain. Associated with non-repaired capsulotomy

Femoroacetabular impingement has also been implicated in the development of hip instability, and the concept of FAI-induced instability has been recently described. Philippon et al. report evidence of FAI in 9/14 (64%) in football players treated for posterior hip subluxation. In addition, a study by Krych et al. demonstrated radiographic evidence of FAI in 81% of patients that presented with a posterior acetabular lip fracture following subluxation (33). Of these, 45% had evidence of a CAM deformity, while 55% had both CAM and pincer deformities. FAI-induced instability differs from traumatic hip dislocations, as these are lower energy injury on the athletic playing fields. Krych and colleagues proposed that the mechanism of injury is a result of hip flexion, and internal rotation creates abnormal contact between the CAM lesion and the anterior acetabulum, which would then lever the femoral head posteriorly (33).

The treating hip arthroscopist should be aware of hip atraumatic microinstability in the borderline dysplastic patient or patient with generalized ligamentous laxity. Acetabular dysplasia is defined by a lateral center edge angle (LCEA) of <20° and Tönnis angle >12° with borderline dysplastic patients having LCEA angles between 20° and 25° (Figures 3C,D). Hip dysplasia results in undercoverage of the femoral head by the acetabulum, which alters hip joint biomechanics, placing additional stress on the labrum, anterior capsule, and dynamic stabilizers (34, 35). These hips force the dysplastic and borderline patients to rely on the hip soft tissue stabilizers (cartilage, labrum, and capsule) for stability of the hip through the full range of motion. Notably, the iliocapsularis has been found to hypertrophy in dysplastic patients, with a recent imaging study reporting that the ratio of the iliocapsularis to the rectus femoris can be a subtle marker of instability in this cohort (14, 36). This marker may help aid the hip arthroscopist in determining whether symptoms are resulting from the instability of dysplasia, or impingement from cam deformities in patients presenting with radiographic signs of both dysplasia and impingement (36). While true dysplasia is generally managed with periacetabular osteotomy, borderline dysplasia has been treated arthroscopically with conflicting results (35, 37, 38). In a recent study of 22 patients with borderline dysplasia, the authors report good outcomes for patients that underwent arthroscopic labral preservation and repair with capsular plication (35). Capsular management is especially critical in patients with borderline dysplasia, as iatrogenic injury to the capsule without appropriate repair will destabilize the hip joint (34, 35). Additionally, more overt atraumatic microinstability has been described in patients that have generalized capsular laxity (39). Capsular laxity arises secondarily to connective tissue disorders, such as Ehlers Danlos and Marfan syndromes, but can also be seen in patients subjected to repetitive microtrauma (25, 32). While previously managed by thermal capsulorrhaphy, capsular laxity is currently addressed through suture-based plication techniques (2, 39). Microtrauma-associated hip instability remains an evolving topic of interest, as it contains elements of both traumatic and atraumatic hip instability (32, 40).

There have been at least eight published case reports of gross dislocation after hip arthroscopy (41–48). While rare, iatrogenic hip instability is a feared and devastating complication (42, 43, 46). Risk factors for postoperative instability include an open capsulotomy without repair, as well as patients having acetabular dysplasia, hypermobility, or ligamentous laxity (19, 24, 49). It is thought that the number of cases of macroinstability (hip dislocations) is underreported; however, there is a group of patients with iatrogenically induced microinstability that may be much more common and unrecognized after hip arthroscopy. McCormick and colleagues reported on 25 patients that required revision surgery over a 1-year period, and 16 of the 25 patients had residual FAI that necessitated revision surgery. The remaining nine patients had capsular abnormalities on magnetic resonance arthrography (MRA), and seven of nine had capsular defects that required revision surgery to repair the non-healing portions of the capsule (50).

## Surgical Technique

### Capsulotomy

**Figure 4:**
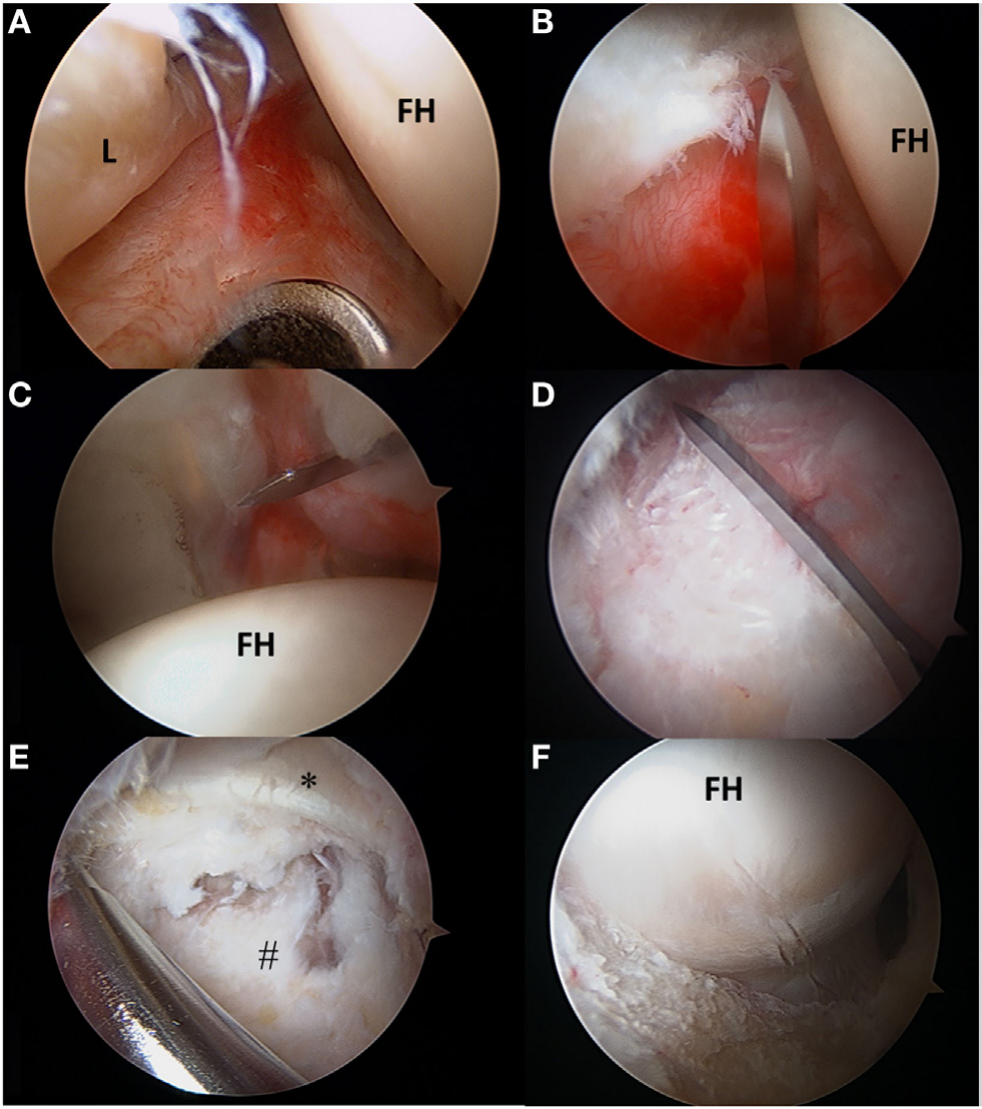
**(A–F)** Transverse and T-type capsulotomy. **(A)** The anterolateral portal is seen penetrating the capsule with the scope viewing through the mid-anterior portal. **(B)** The interportal capsulotomy as seen through the mid-anterior portal. The capsulotomy must begin at least 5 mm from the labrum to ensure adequate tissue for repair. **(C)** Complete interportal capsulotomy to a final length of 2–4 cm depending on the central compartment pathology. **(D)** To view the peripheral compartment, a T-capsulotomy is performed along the ILFL perpendicular to the interportal capsulotomy between the gluteus minimus and iliocapsularis. **(E)** The ILFL leaflets (^#^) and the reflected head of the rectus femoris (*) can be visualized in proximity to the T- capsulotomy. **(F)** The T-capsulotomy extends down the femoral neck to expose the CAM deformity. FH, femoral head; L, labrum.

With the substantial increase in hip arthroscopy over the past decade, several different techniques, to both incise and repair the capsule, have been described. These techniques include capsulectomy, extensile interportal capsulotomy with or without repair, or a T-capsulotomy with partial or complete repair. Once AL portal and modified-anterior portal (MAP) are established, a transverse interportal capsulotomy is performed 5–10 mm from the labrum, running between 11:00 and 2:00 measuring approximately 2–4 cm depending on the location of the pathology (Figures 4A–C) (2, 19, 49, 51). A blade is generally preferred to a radiofrequency ablator to minimize the risk of iatrogenic labral and cartilage injury while also making capsular closure more precise, if warranted (2, 19, 49). Once the chondrolabral pathology has been treated, the instruments are removed from the central compartment, and the traction is suspended flexing the hip approximately 30°. Some surgeons prefer a T-capsulotomy by extending the interportal capsulotomy distally at its midpoint through a distal anterolateral accessory (DALA) portal (Figure 4D). In this case, it is critical to identify the intercapsular plane between the two limbs of the ILFL located between the attachment sites of the gluteus minimus and ilocapsularis. Correct identification of this plane will facilitate capsulotomy, as the medial capsule will retract with the iliocapsularis and the lateral capsule will retract with the gluteus minimus (2). Advantages of the T-capsulotomy include improved access in the peripheral compartment and visualization of the head–neck junction for cam deformity correction (Figures 4E,F) (2, 49). The capsular suspension technique can facilitate visualization by placing horizontal mattress traction sutures through the medial and lateral leaflets of the ILFL. These stitches are clamped outside the portals with a hemostat to elevate the leaflets for improved visualization, and their closure facilitates a tension-free repair (52). A limited or focal capsulectomy may provide advantages in cases of capsular hypertrophy or stiffness, but this comes at the expense of permanently altering hip joint biomechanics and likely imposes an as yet undefined degree of instability (2, 19, 24, 49). A recent survey of 27 high-volume hip arthroscopists found that they uniformly prefer capsulotomy over capsulectomy (53).

### Capsular Repair and Plication

Capsular repair is growing in popularity, particularly in cases of capsular incompetence, atraumatic instability, or hyperlaxity. In a cross-sectional survey, Gupta et al. explained that only 11% of high-volume hip arthroscopists never close the capsule compared to 48% that close the capsule >50% of the time (53). Seventy-eight percent of these surgeons decided whether or not to close the capsule based on the risk for instability conditions and intraoperative findings. Capsular repair techniques are varied based on size, type, location of the capsulotomy as well as surgeon preference.

**Figure 5:**
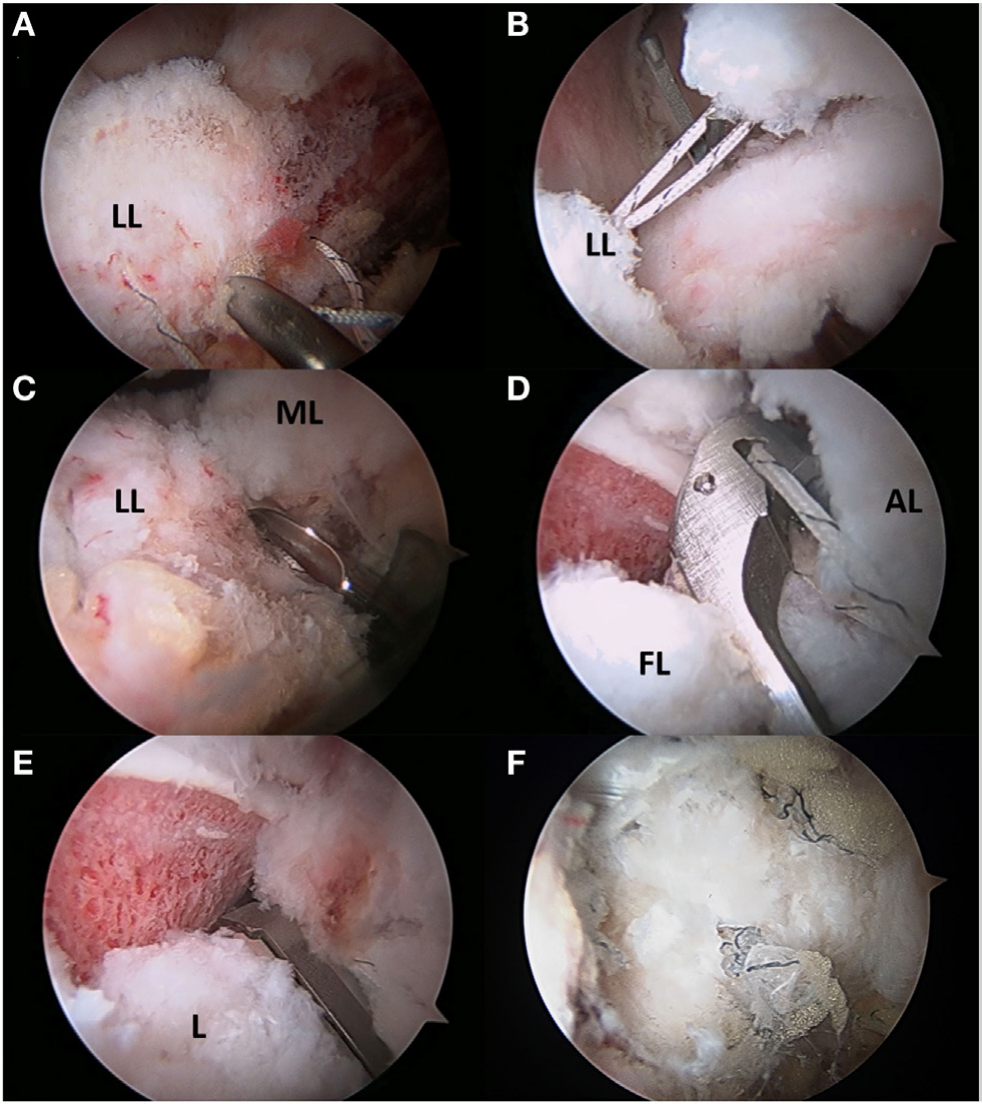
**(A–F)** Capsular repair. **(A)** Capsule repair is initiated by using a tissue penetrating device to pass suture through the lateral leaflet of the ILFL. **(B)** Suture is then passed through the medial leaflet of the ILFL **(B)**, and a knot is tied after each successive stitch has been passed **(C)**. The interportal capsulotomy is repaired by passing suture through the acetabular side of the ILFL **(D)** and femoral side of the ILFL **(E)**. The repaired capsule visualized through the mid-anterior portal.

Harris et al. described a technique to employ an InJector II suture passer (Stryker Sports Medicine, Greenwood Village, CO, USA) for closing the capsulotomy through a single portal and for complete closure of both limbs of the T-capsulotomy (49). In the case of the T-capsulotomy, the vertical arm is closed distally to proximally, starting at the base of the ILFL using a suture shuttling technique (Slingshot, Stryker Sports Medicine, Greenwood Village, CO, USA). With the arthroscope in the MAP, an 8.5 mm cannula is placed in the AL and the DALA portals. The Slingshot is placed through the AL portal to penetrate the lateral ILFL (Figure 5A), and the suture is retrieved using the Slingshot through the DALA portal (Figure 5B). Via the DALA portal, a suture retriever is used to grasp the suture from the AL portal to allow for arthroscopic knot tying (Figure 5C). Capsular plication or capsulorrhaphy can be considered to limit capsular redundancy (19, 54). Capsular plication is performed with the hip in 45° flexion, so that side-to-side stitches take larger bites to reduce extraneous capsular elements and decrease the capsular volume (2). Once the vertical limb of the T-capsulotomy is closed, the interportal capsulotomy can be closed with two to three sutures using the InJector II or Slingshot. The posterolateral extent of the interportal capsulotomy is closed though the AL portal. The suture is passed through the acetabular side of the ILFL and then the femoral side of the ILFL. The anteromedial extent of the interportal capsulotomy is closed through the DALA portal using similar steps (Figures 5D,E). The authors’ preference is to pass the sutures before tying in order to facilitate proper visualization, then the sutures can be tied sequentially until the capsule is closed entirely (Figure 5F).

## Clinical Outcomes

When indicated, arthroscopic correction of FAI has produced high functional outcomes over the short- and mid-term (Table 2). While it has generally proven successful for the treatment of FAI, further research is required to assess the utility of arthroscopy in the setting of hip dysplasia, preexisting osteoarthritis, and cartilage damage (35, 55–58). Exposure of the cam and pincer deformities is another limitation of hip arthroscopy. This requirement often necessitates a capsulotomy to ensure adequate visualization of the offending pathology. Given the variation in capsulotomy and capsular repair techniques, recent research has focused on the clinical outcomes as they relate to differences in capsular management. A recent review found that unrepaired capsulotomy may be preferred for patients with preoperative stiffness, rheumatologic conditions, or synovial proliferative disorders, such as pigmented villonodular synovitis (PVNS) (19). Another recent study evaluated 2-year patient-reported outcome scores (PROs) in 168 patients with and 235 patients without capsular repair. The authors found that the Hip outcome score-activities of daily living (HOS-ADL) and non-arthritic hip scores (NAHS) improved significantly in the capsular repair group compared to the non-repair group (59). They reported that patient age, gender, and the extent of chondral damage were predictive of the capsular management strategy (59). In contrast, another recent study showed improved outcomes for patients who received complete rather than partial repair of a T-type capsulotomy (51). In this study, Frank et al. compared 32 partial repairs of just the vertical arm of the T-capsulotomy with 32 complete repairs of both the vertical and horizontal arms. The authors found that patients with complete repair had improved Hip Outcome Score-Sports Specific subscale (HOS-SS) at the 6-month, 1-, and 2-year time points. Additionally, the patients in the partial repair group had a higher revision rate at 13%, compared to 0% in the complete repair group. Nevertheless, preoperative to postoperative PROs improved for all groups of patients in both studies. The initial clinical studies suggest that complete capsular repair can improve hip functional outcomes and return to athletic activity. Moreover, there appears to be a higher revision rate in cases in which the hip capsule is not repaired completely (50). Finally, the importance of hip capsular stability to overall clinical outcome was elegantly illustrated by examining a patient cohort that was painful following index hip arthroscopy without capsular closure (60). Wylie and colleagues performed revision hip arthroscopy with routine capsular closure on this patient cohort and demonstrated significant improvements in all PROs at >2 years of follow-up. While these clinical outcome studies are not without limitations, the overall body of literature to date demonstrates the importance of capsular stability to clinical outcomes following hip arthroscopy.

**Table 2:** **Outcomes of hip arthroscopy for FAI**.

Reference	Design	Patients (hips)	Follow-up (months)	Functional outcome scores
Ilizaliturri et al. (71)	Retrospective case series	13 (14)	30	9.6 point increase in WOMAC
Philippon (61)	Retrospective case series	112	28	24 point HHS increase, median satisfaction 9/10
Byrd and Jones (68)	Retrospective case series	200 (207)	16	20 point HHS increase, 1.5% complication rate
Larson and Giveans (72)	Retrospective cohort–control	76	21	Higher 1-year HHS scores in labral refixation (94.3) compared to debridement (88.9) groups (*p* < 0.01)
Schilders et al. (66)	Retrospective cohort–control	96 (101)	29	Higher improvement in 2-year HHS scores in labral refixation (33) compared to labral debridement (26) (*p* = 0.034)
Malviya et al. (62)	Retrospective case series	612	38	Quality of Life increase from 0.946 to 0.974 (*p* < 0.001)
Skendzel et al. (67)	Retrospective cohort–control	323	73	Average HHS, HOS-ADL, ad HOS-SS scores increased significantly from preoperative values. Patients with joint space >2 mm had higher increases in HOS-ADL (15 vs. −6; *p* = 0.035) and HOS-SS (34.8 vs. 3.6; *p* = 0.005)
Frank et al. (51)	Retrospective cohort–control	64	30	Average HHS, HOS-ADL, ad HOS-SS scores increased for significantly from preoperative values (*p* < 0.001). Patients with full T-capsulotomy repair had higher HOS-SS outcome scores (83.6 vs. 87.3; *p* = 0.001) than partially repaired capsulotomy
Domb et al. (59)	Retrospective cohort–control	403	24	Average HHS, HOS-ADL, ad HOS-SS scores increased for significantly from preoperative values (*p* < 0.001). No differences in HHS, HOS-ADL, HOS-SS, and NAHS for patients with repaired vs. unrepaired capsulotomy

Hip arthroscopy is an emerging field, and additional basic science, translational, and clinical research is required to provide both insight into the natural history of the disease as well as continue to improve patient outcomes. Currently, the state of the literature remains limited to small to medium sized case series reporting short to medium term outcomes. To date, there are no published randomized controlled trials evaluating operative vs. non-operative management for FAI. As the rates of hip arthroscopy have increased substantially over the past decade, ongoing investigations into patient clinical and functional outcomes are required to justify the increase in case volume. At this point, numerous studies have demonstrated that hip arthroscopy, when indicated, is successful at relieving patient pain and improving both patient-reported clinical outcomes as well as return to activity and sport in cohorts of elite and recreational athletes (Table 2) (61–68). Further, several studies have shown that arthroscopic surgery on non-arthritic patients with FAI is cost-effective when compared to observation (69, 70). Capsular management remains one of the many topics in the field of hip arthroscopy that is continually evolving. Additional investigation into capsular biomechanics, alternate closure techniques, and long-term patient outcomes is required to further develop the fund of knowledge surrounding capsular management in hip arthroscopy.

## Conclusion

Hip arthroscopy for the treatment of chondrolabral pathology as well as FAI has been growing exponentially. The structure and function of the hip joint capsule is not well understood. There have been recent scientific studies that suggest that a capsulotomy may affect the ability to maintain normal hip translation, rotation, and axial strain, and therefore, the hip may become unstable due to altered hip joint kinematics. Clinical outcomes after hip arthroscopy also suggest a more predictable and reliable hip function with complete capsular repair with a lower rate of revision surgery. The modern strategy of stabilization of chondrolabral pathology, comprehensive treatment of FAI, and complete capsular repair appear to show pain relief, improvement in activities of daily living, the ability to return to athletic activity, and minimize revision surgery.

## Author Contributions

All authors listed, have made substantial, direct and intellectual contribution to the work, and approved it for publication.

## Conflict of Interest Statement

AB: paid consultant Arthrex. Publishing royalties: SLACK incorporated, Springer. RM III: paid consultant KNG Health Consulting; Pivot Medical, Smith & Nephew; Stryker. MS: paid consultant: Smith & Nephew. SN: paid consultant: Ossur, Stryker. The remaining coauthors declare that the research was conducted in the absence of any commercial or financial relationships that could be construed as a potential conflict of interest.
